# Has the matching between urban population aging and older adult care facilities achieved coupling coordination?—An empirical analysis based on spatiotemporal evolution and multifactor interaction mechanisms

**DOI:** 10.3389/fpubh.2025.1644849

**Published:** 2025-07-22

**Authors:** Q. Liu, S. L. Zhao, Y. F. Li

**Affiliations:** ^1^College of Resources and Environmental Engineering, Tianshui Normal University, Tianshui, China; ^2^State Key Laboratory of Soil Erosion and Dryland Farming on the Loess Plateau, Institute of Soil and Water Conservation, Chinese Academy of Sciences, Ministry of Water Resources, Yangling, China

**Keywords:** population aging, older adult care facilities, coupling coordination, spatial matching, Gansu Province

## Abstract

The aging population has become a global socio-economic challenge, particularly in economically underdeveloped regions, where it profoundly impacts the allocation of medical resources, social security systems, and land use patterns. However, existing research has paid insufficient attention to the coordination between population aging and the spatial distribution of older adult care facilities, making it difficult to provide scientific evidence for optimizing regional older adult care resources. This study explores the temporal and spatial evolution characteristics of aging and its relationship with older adult care facilities based on data from Gansu Province from 2000 to 2020. Using ArcGIS for spatial visualization analysis, we measure matching equity through the Gini coefficient and assess the coupling coordination relationship using a coupling coordination degree model. Additionally, we employ geographic detectors to identify key influencing factors. The findings indicate that the level of aging in Gansu Province continues to rise with significant spatial heterogeneity; northern and central regions experience accelerated aging, while southern areas show slower growth rates. The distribution of older adult care facilities is highly uneven; economically developed cities like Lanzhou concentrate resources, while severely underdeveloped areas in the north and west lack adequate facilities. Fluctuations in the Gini coefficient suggest that equity in matching between aging populations and older adult care facilities has not consistently improved over time; overall coupling coordination levels remain low. Geographic detection results reveal that population density, economic development, fiscal investment, and digital financial development are critical factors affecting matching degrees among these elements. Notably, interaction effects between population density and foundational aspects of aging are most pronounced (*q* = 0.7498). This study deepens our understanding of the relationship between population aging and older adult care facility matching dynamics while providing a scientific basis for optimizing layout strategies for eldercare infrastructure and land use planning in underdeveloped regions. It emphasizes the necessity of tailored approaches to enhance equity in eldercare resource allocation according to local conditions.

## 1 Introduction

Population aging has become an increasingly prominent global issue, particularly in China, where rapid economic development and declining fertility rates have contributed to a growing aging population (1). According to data from the China Statistical, China has experienced a significant aging process over the past few decades (2). By 2030, individuals aged 65 and above are projected to account for nearly 20% of the total population (3). This trend presents varying challenges across regions, with western China experiencing faster and more severe aging compared to the rest of the country (4). Gansu Province, as a representative province in the northwest, faces the dual challenges of abundant natural resources and lagging economic development (5), while its aging process is accelerating, posing urgent social challenges (6).

Gansu Province, located in China’s northwestern inland region, is endowed with abundant natural resources, but its geographical conditions and economic structure make it particularly vulnerable in addressing population aging. The aging issue in Gansu is not only reflected in the overall increase in the older adult population but also in the significant regional disparities in aging processes as well as older adult care facilities. In particular, the northern and central regions, such as Jiuquan and Zhangye, exhibit rapid aging due to relatively stronger economic development. In contrast, the southern and western regions, including Longnan and Gannan Tibetan Autonomous Prefecture, face slower aging but still struggle with a growing older adult population due to less developed economies and insufficient older adult care infrastructure.

The theoretical foundation of this study is built upon the interdisciplinary integration of spatial justice theory, health equity theory, and social resource allocation theory. Spatial Justice Theory emphasizes the fairness of spatial distribution of social resources and service facilities, arguing that all populations, regardless of their geographical location, should enjoy equal rights to access basic public services. In the field of older adult care services, spatial justice means that older adult populations should receive corresponding older adult care facility allocation based on their spatial distribution, avoiding service inequities caused by geographical location differences. Health Equity Theory further emphasizes that health-related resource allocation should be based on need rather than ability to pay, with particular attention to health rights protection for vulnerable groups (30, 31). For older adult populations, health equity encompasses not only accessibility of medical services but also multi-dimensional health promotion resources including older adult care, social support, and other services. Social Resource Allocation Theory approaches from the dual perspective of efficiency and equity, emphasizing how to achieve social welfare maximization through optimized allocation under resource-constrained conditions (32, 33). The integration of these three theories provides an analytical framework for this study: (1) Spatial justice theory guides our focus on the fairness of spatial distribution of older adult care facilities, employing the Gini coefficient to quantify spatial inequity; (2) Health equity theory directs us to consider older adult populations’ care needs as the core basis for resource allocation, rather than focusing solely on economic benefits; (3) Social resource allocation theory provides theoretical support for seeking balance between efficiency and equity under resource constraints, guiding the construction of coupling coordination degree models and policy recommendation formulation. Therefore, this study essentially explores how to achieve spatial justice and health equity in older adult resource allocation in western underdeveloped regions, providing theoretical guidance and practical pathways for constructing a more just and sustainable older adult care service system.

Studies on population aging have mainly focused on several aspects: the spatial distribution of aging, the impact of aging on social services and healthcare systems (7), and the matching between aging populations and older adult care facilities (8). Early studies primarily concentrated on the national level, exploring the overall aging process in China and its macroeconomic impacts. For example, Lin et al. (9) and Liu et al. (10) pointed out that China’s aging problem is primarily reflected in the decline of the working-age population and the rising proportion of older adult individuals, which will have profound impacts on the labor market, social security system, and healthcare resource allocation. However, most existing studies focus on large cities or economically developed areas, with limited research on underdeveloped western regions, such as Gansu Province. The marginal contributions of this study are threefold: First, unlike existing studies that primarily focus on older adult care facility allocation in developed urban plains, this research concentrates on topographically complex and economically underdeveloped western mountainous areas, filling the research gap for such regions. Current research mainly focuses on eastern developed cities such as Beijing, Shanghai, and Shenzhen, with relatively insufficient attention to western mountainous areas. Second, this study establishes a facility-population spatial matching evaluation framework applicable under resource-constrained conditions, breaking through the limitations of traditional Euclidean distance calculation methods by adopting network analysis and terrain resistance coefficient-corrected accessibility models to reflect actual service capacity. Finally, the research proposes hierarchical facility layout optimization strategies, providing replicable policy tools for similar regions.

In recent years, there has been an increasing focus on regional differences in aging processes. Wei e al. (11) examined the temporal and spatial variations in aging across China, highlighting that economic development, social security systems, and healthcare resource distribution are key factors affecting regional aging. Zhang et al. (12) further explored the relationship between aging and social welfare facilities in western China, suggesting that the accelerated aging process and the uneven distribution of older adult care facilities lead to significant challenges, particularly in economically underdeveloped regions, where the challenges of aging societies are more severe. Comparative analysis reveals significant differences in spatial allocation of older adult care facilities between developed and underdeveloped regions. According to the China Aging Industry Development Report (2022), developed regions typically achieve facility density of 3.5–4.2 beds per 1,000 older adult, with relatively balanced spatial distribution, mainly focusing on service quality optimization. However, our study finds that Gansu Province’s facility density is only 1.8 beds per 1,000 older adult, a gap of over 60%, with extremely unbalanced spatial distribution where 73.2% of facilities are concentrated in county-level cities and above, while rural areas suffer from severe coverage inadequacy. The average service radius in developed regions is 2–3 kilometers, whereas in Gansu’s mountainous areas, the effective service radius expands to 8–12 kilometers, with some remote townships exceeding 20 kilometers.

Existing research exhibits three significant knowledge gaps: First, at the methodological level, most studies employ simple Euclidean distance calculation methods to assess older adult care facility accessibility, ignoring the impacts of topographical complexity, transportation network constraints, and actual travel costs. For example, in mountainous and remote areas, facilities with straight-line distances of only 2–3 kilometers may require traversing complex terrain for actual accessibility, extending service radii to 10–15 kilometers. Traditional methods severely underestimate such accessibility barriers. Second, at the regional research level, existing studies primarily focus on eastern developed cities (such as Beijing, Shanghai, and Shenzhen in plain areas), with severely insufficient attention to western underdeveloped mountainous areas. According to literature statistics, over 80% of relevant studies focus on developed regions with per capita GDP exceeding 100,000 yuan, while studies targeting regions with per capita GDP below 50,000 yuan and complex terrain account for less than 15%. This causes existing theoretical frameworks to be difficult to apply to underdeveloped areas with both resource and geographical constraints. Third, at the theoretical framework level, there is a lack of facility-population spatial matching evaluation frameworks applicable under resource-constrained conditions. Existing models are mostly constructed based on resource-abundant, transportation-convenient developed regions, assuming that facility supply can rapidly respond to demand changes. However, this assumption is clearly invalid in underdeveloped areas with limited fiscal capacity and weak infrastructure. This study addresses the above knowledge gaps through the following innovations: (1) Methodological innovation: Constructing a terrain resistance coefficient-corrected network accessibility model that incorporates terrain slope, road grade, and transportation convenience into accessibility calculations, more accurately reflecting actual service capacity in mountainous areas; (2) Regional research gap filling: Taking Gansu Province as a representative western underdeveloped mountainous area as the research object, filling the research gap for such regions and providing a referenceable theoretical framework for similar areas; (3) Theoretical framework construction: Establishing a spatial matching evaluation system applicable under resource-constrained conditions and proposing hierarchical facility layout optimization strategies, providing practical tools for older adult care facility planning in underdeveloped areas.

However, there are still several gaps in the existing literature. First, most studies focus on economically developed regions, especially coastal cities, with relatively little attention paid to the aging processes in western regions and their relationship with social service facilities (13). Second, while some studies have explored the pressures of aging on social services, there is still a lack of effective analytical frameworks for accurately assessing the alignment between aging populations and older adult care facilities at the regional level (14). Finally, although some scholars have identified the multidimensional factors influencing regional aging, limited research has examined how these factors interact and influence the provision of older adult care services (15).

This study focuses on analyzing the spatial and temporal evolution of population aging and older adult care facilities in Gansu Province from 2000 to 2020. By utilizing ArcGIS and the natural breakpoint method, combined with data from 14 prefecture-level cities, this study identifies the distinct patterns of aging in various regions and explores the gaps in older adult care services. The results aim to provide theoretical support for Gansu’s response to aging, guide the construction of older adult care facilities, and inform regional development planning. This research is not only significant for addressing the aging problem in Gansu but also offers valuable insights for other western provinces in China. By analyzing the spatial and temporal evolution of aging and older adult care facilities, this study hopes to provide policymakers with data-driven support and a more comprehensive understanding, ultimately aiding local governments in optimizing resource allocation and strengthening their capacity to cope with the challenges of population aging.

## 2 Materials and methods

### 2.1 Study area

**Figure 1:**
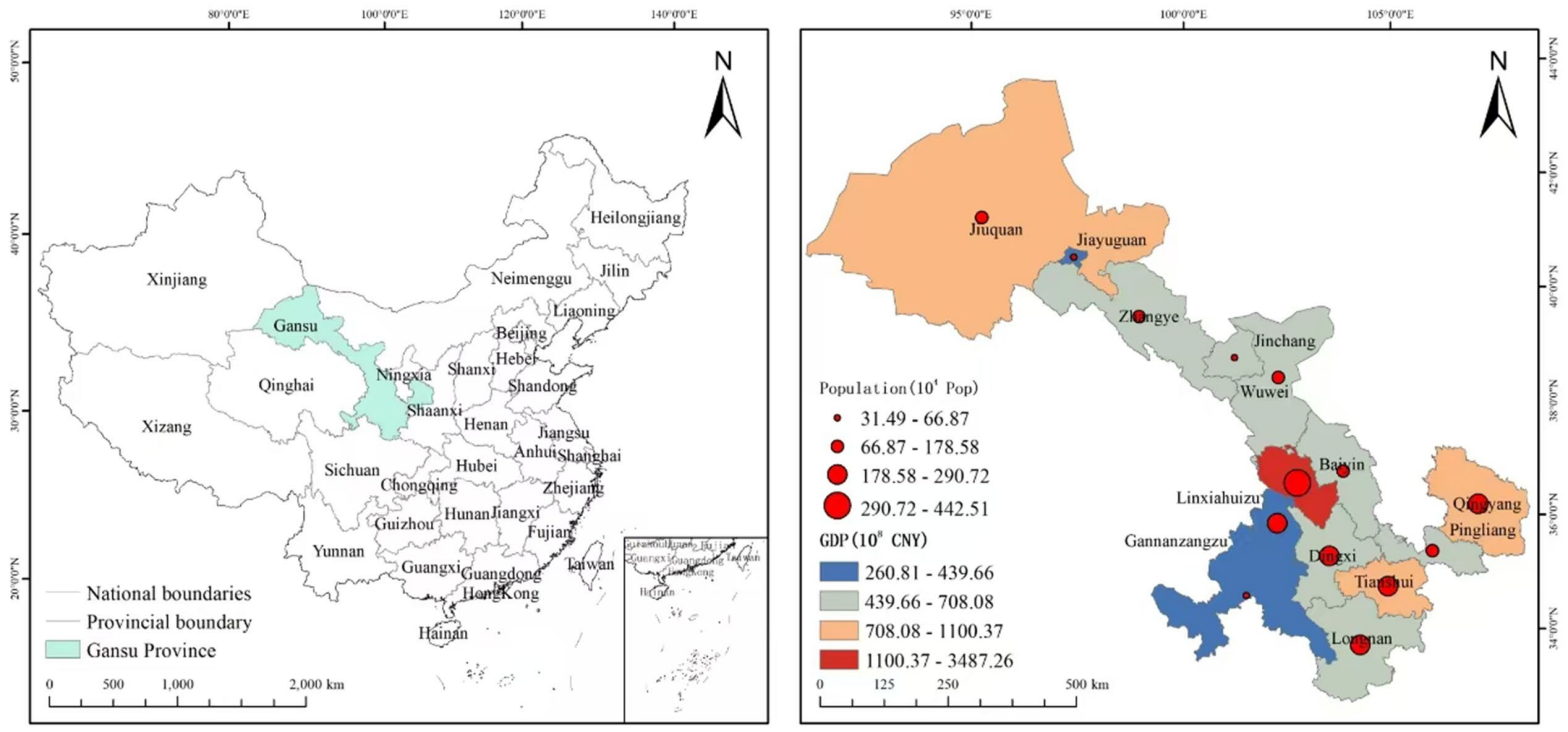
Schematic map of the study area location.

Gansu Province is located in northwest China, situated at the junction of the Loess Plateau, Qilian Mountains, and Hexi Corridor, occupying an important strategic position in both the ancient Silk Road and the modern “Belt and Road Initiative.” The province is located between 32°11′–42°57′N latitude and 92°13′–108°46′E longitude, bordered by Shaanxi Province to the east, Sichuan Province to the south, Qinghai Province to the west, and Inner Mongolia Autonomous Region to the north. Gansu Province covers an area of approximately 454,000 square kilometers, making it one of the largest provinces in northwest China, and administers 14 prefecture-level administrative units: Lanzhou (provincial capital), Jiuquan, Zhangye, Jiayuguan, Wuwei, Jinchang, Baiyin, Tianshui, Wushan, Pingliang, Qingyang, Longnan, Dingxi, Linxia Hui Autonomous Prefecture, and Gannan Tibetan Autonomous Prefecture. Lanzhou serves as the major urban center, transportation hub, and industrial base, benefiting from relatively favorable terrain, better transportation connectivity, and concentrated government investment. The northern region of Gansu Province, including Jiuquan, Zhangye, Jiayuguan, Wuwei, and Jinchang, benefits from abundant energy resources such as coal, oil, and natural gas, modern agriculture, and strategic locations along major transportation corridors. The southern and western regions of Gansu Province, including Longnan, Gannan, and Linxia, are characterized by mountainous terrain, ethnic diversity, and traditional agricultural economies, with slower economic development and limited infrastructure. The population of approximately 24.58 million in Gansu Province in 2022 is extremely unevenly distributed, with population density varying dramatically from over 200 people per square kilometer in urban areas like Lanzhou to less than 10 people in western desert regions. Major population centers include the Lanzhou metropolitan area with over 4 million people, Tianshui City with over 3 million people, and medium-sized cities in the Hexi Corridor with populations between 1–2 million. The province exhibits significant economic heterogeneity that directly affects older adult care infrastructure development: Lanzhou, Jiuquan, and Jiayuguan benefit from energy industries and government investment; moderately developed areas like Tianshui, Zhangye, and Wuwei rely on agriculture and regional services; while less developed areas including Longnan, Gannan, and Linxia depend primarily on subsistence agriculture but have limited fiscal capacity for social infrastructure investment. According to the Seventh National Population Census (2020), people aged 60 and above account for 19.06% of the total population. The aging patterns reflect distinct regional characteristics influenced by population mobility, where economically developed areas attract young workers but subsequently experience rapid demographic transitions, while underdeveloped areas face youth population outflow, accelerating the local aging process (see Figure 1).

### 2.2 Data sources

**Figure 2:**
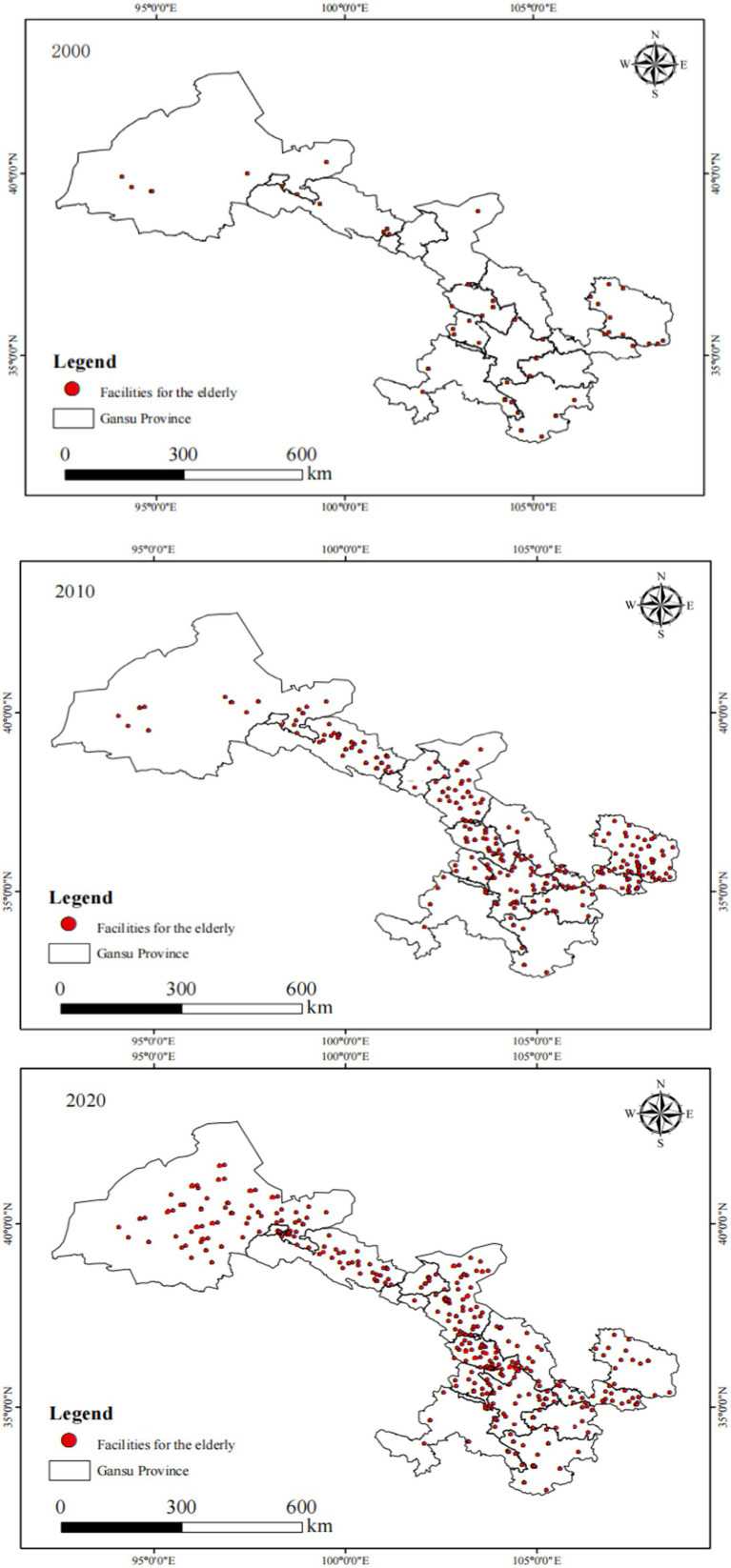
Spatial distribution map of older adult care facilities.

The population data of individuals aged 65 and above for each spatial unit in Gansu Province is sourced from the fifth (2000), sixth (2010), and seventh (2020) national censuses, which indicate that the proportion of the older adult population has been increasing over the past two decades. According to the regulations on the management of older adult care institutions issued by the ministry of civil affairs of China, older adult care facilities can only begin offering services after they have registered with the relevant market supervision authorities. Based on this, a comprehensive list of registered older adult care facilities has been compiled as of 2020 by accessing the website of the Gansu Provincial Department of Civil Affairs. This list includes information such as addresses, types of institutions (privately operated/government-operated), and registered bed capacities for each facility. The address information was converted into geographic coordinates (latitude and longitude) to serve as spatial data for these facilities, which were subsequently categorized into their respective spatial units. Additionally, through searches and visits to various older adult care facility websites, their establishment dates were confirmed; these dates were then classified according to three temporal nodes corresponding to the census years: 2000, 2010, and 2020 (Figure 2). It is important to note that the older adult care facility data in this study has a clearly defined scope. According to the regulations on the management of older adult care institutions of the People’s Republic of China, our dataset only includes older adult care institutions formally registered with the Gansu Provincial Department of Civil Affairs, specifically including: (1) Government-invested public nursing homes and older adult care homes; (2) Private nursing homes and care facilities established by social forces; (3) Professional older adult care service institutions with formal business licenses and service permits. This study does not include the following types of older adult care service facilities: (1) Community day care centers and older adult care centers; (2) Home-based older adult care service stations; (3) Informal village-level older adult care mutual aid points; (4) Non-specialized older adult care portions within medical-care integration institutions; (5) Other unregistered older adult care service facilities. This data selection is based on the following considerations: First, data from registered institutions have higher availability, accuracy, and comparability, facilitating standardized spatial analysis; second, these institutions provide relatively standardized and professional older adult care services, better reflecting the spatial allocation of formal older adult care service systems; third, registered institutions typically have clear service capacity and service scope, facilitating quantitative analysis.

### 2.3 Research methodology

#### 2.3.1 Integration method of topographic elements in network analysis

To accurately reflect the impact of Gansu Province’s complex terrain on older adult care facility accessibility, this study integrates three topographic elements—slope, elevation, and road type—into network analysis:

1. Topographic element data preprocessing

Slope element: Based on 30 m resolution DEM data, slope information is extracted using ArcGIS Slope tool and classified by the following rules: 0–5° (flat, resistance coefficient 1.0), 5–15° (gentle slope, 1.2), 15–25° (steep slope, 1.5), 25–35° (sharp slope, 2.0), >35° (precipitous slope, 3.0).

Elevation element: Considering the impact of high altitude on transportation and service provision, resistance coefficients are set as: <1,500 m (1.0), 1,500-2500 m (1.2), 2,500-3500 m (1.5), >3,500 m (2.0).

Road type: According to Gansu Province road grade standards, speed and resistance parameters are set: Highway (120 km/h, resistance 0.8), National road (80 km/h, 1.0), Provincial road (60 km/h, 1.2), County road (40 km/h, 1.5), Rural road (20 km/h, 2.0).

1. Comprehensive resistance surface construction

A comprehensive terrain resistance surface is constructed using weighted overlay method: Comprehensive Resistance Value = Slope Resistance×0.4 + Elevation Resistance×0.3 + Road Resistance×0.3.

1. Network analysis implementation steps

① Import road network data into ArcGIS Network Dataset; ② Assign corresponding comprehensive resistance values as travel costs for each road segment; ③ Use Service Area tool to calculate accessibility using resistance values instead of traditional distance; ④ Generate isochrones and service ranges considering terrain resistance.

#### 2.3.2 Gini coefficient

Based on the population aged 65 and above in various spatial units and the accessibility indicators of older adult care facilities, this study employs the Gini coefficient to comprehensively evaluate the matching relationship between the aging population and older adult care facilities at three different time points from a holistic perspective (16). The calculation formulas used in this study are shown in Equations 1–6:


(1) $$G=1-\sum_{i=1}^{n}(P_{i}-P_{.})(S_{i}-S_{.})$$

In the equation: *G* represents the Gini coefficient; *P_i_* denotes the cumulative proportion of the older adult population aged 65 and above in spatial unit i; *S_i_* refers to the accessibility variable of older adult care facilities in spatial unit i; where i is the identifier for spatial units, with *i* = 1, 2, …, n.

#### 2.3.3 Coupled coordination model

The coupling coordination degree is utilized to characterize the compatibility between the older adult population and eldercare facilities. Based on the number of individuals aged 65 and above in each spatial unit, as well as accessibility indicators for eldercare facilities, a coupling coordination model is employed to measure the compatibility index at three time points (16). This analysis adopts a localized perspective to examine the spatiotemporal differentiation characteristics of the matching relationship between the older adult population and eldercare facilities (17). The calculation formula is as follows:


(2) $$C=2\sqrt{\frac{E_{i}\times S_{i}}{{\left(E_{i}+S_{i}\right)}^{2}}}$$

(3) $$T=\alpha \times E_{i}+\beta \times S_{i}$$

(4) $$D=\sqrt{C\times T}$$

In the formula: *E_i_* represents the standardized range value of the older adult population aged 65 and above in spatial unit i; C denotes the coupling degree, which is determined by the values of two subsystems: older adult population *Ei* and Senior Care Facilities (*S_i_*); T refers to a comprehensive evaluation index for accessibility between the older adult population and Senior Care Facilities, reflecting their overall synergistic effect or contribution; based on existing research, this study proposes that both subsystems are equally important in measuring matching relationships, thus setting *α* = *β* = 0.5; *D* indicates the coupling coordination degree, which reflects the level of coordinated interaction between the older adult population and Senior Care Facilities. The value of *D* ranges from 0 to 1; a higher value signifies a greater degree of orderly coordination between these two subsystems.

#### 2.3.4 Geographical detector

The Geographic Detector is an algorithmic program designed to explore the interactions between explanatory variables and spatial differentiation (18). It can be applied across various fields, including social sciences and natural sciences (19). This study aims to investigate the relationship between coupling coordination degree and factors such as population, society, economy, and natural factors (20). The calculation formula is as follows:


(5) $$q=1-\frac{1}{N\sigma^{2}}\sum_{h=1}^{L}N_{h}\sigma_{h}^{2}$$

In the context of Meta-analysis, the *q* statistic, which ranges from 0 to 1, is used to assess the heterogeneity among studies. A higher q value suggests a greater degree of heterogeneity, indicating that the factor has a more pronounced effect on the coupling coordination degree. This interpretation is supported by the Meta-analysis literature, where the *q* statistic is used to evaluate the variability in effect sizes across studies. The variable *h* signifies the hierarchical levels of the coupling coordination degree, which is a comprehensive indicator reflecting the overall health of a system by considering the dynamic relationships and their impact on the system’s development. *σ* and *N* denote the overall variance and number of units, respectively, while σ_h_ and N_h_ refer to the variance and number of units for city *h* within Gansu Province.

#### 2.3.5 Indicator system development and weight determination

To ensure the reliability of geographic detector analysis results, this study conducted multicollinearity testing for all influencing factors. First, Pearson correlation coefficient matrices among variables were calculated, showing that correlation coefficients among most variables are below 0.7, with only population density and population aging base showing relatively high correlation with a coefficient of 0.726. Variance Inflation Factor VIF was further used for quantitative testing, with the calculation formula


(6) $$\mathrm{VIF}=\frac{1}{1-R^{2}}$$

where *R^2^* is the coefficient of determination for regressing one variable against all other variables. Testing results show: population density *VIF* value of 2.84, population aging base *VIF* value of 3.12, old-age dependency ratio *VIF* value of 1.95, fiscal expenditure *VIF* value of 2.31, digital inclusive finance index *VIF* value of 2.67, number of older adult care institutions per thousand seniors *VIF* value of 1.89, number of healthcare beds per thousand older adult individuals *VIF* value of 2.45, vegetation coverage rate VIF value of 1.76, average annual precipitation *VIF* value of 1.83, PM_2.5_ concentration *VIF* value of 2.18. *VIF* values for all variables are below the critical value of 5, indicating no serious multicollinearity problems among variables, allowing for subsequent geographic detector analysis. For population density and population aging base variables with relatively high correlation, since their VIF values remain within acceptable ranges and theoretically represent different dimensions of population characteristics, they are retained in the analysis model.

#### 2.3.6 Indicator system development and weight determination

The study employs geographic detectors to analyze the factors influencing the interaction between population aging and the availability of older adult care service facilities in Gansu Province. This interaction is complex and influenced by various factors, as detailed in Table 1. Drawing upon existing research and the availability of data, the study focuses on the coupling coordination degree between the aging population and the distribution of older adult care service facilities in the province (21).

**Table 1:** Selection of influencing factors.

Classification	Indicator	Meaning
Population Factors	Population density (R1)	Population density per unit area of land
	Population aging base (R2)	Proportion of population aged 55–64 to total population
	Old-age dependency ratio (R3)	Ratio of older adult population to working-age population
	General public expenditure (J1)	Regional fiscal expenditure on public infrastructure construction
	Digital inclusive finance index (J2)	Reflecting the overall digital finance situation of a region.
Social factors	Number of older adult care facilities per thousand seniors (S1)	(Ratio of nursing homes to total older adult population) × 1,000
	Number of healthcare beds per thousand older adult individuals (S2)	(Hospital bed count to total older adult population ratio) × 1,000
Natural factors	Vegetation coverage rate (Z1)	Forest area as a percentage of total land area
	Average annual precipitation (Z2)	Average annual rainfall is derived from total precipitation over multiple years
	PM_2.5_ (Z3)	Represents atmospheric environmental quality

##### 2.3.6.1 Demographic factors

Population density represents the distribution of the population, specifically highlighting the proportion of individuals aged 55 to 64 across various regions in 2020, which serves as a baseline for population aging. The old-age dependency ratio reflects societal demand for older adult care resources.

##### 2.3.6.2 Economic factors

General public finance expenditure reflects the government’s economic capability to fund public infrastructure projects, while the comprehensive index of digital inclusive finance indicates the stage of digital economy development.

##### 2.3.6.3 Social factors

The ratio of older adult care institutions to every thousand seniors demonstrates the status of eldercare facilities, while the number of healthcare beds per thousand seniors reflects the medical and healthcare conditions.

##### 2.3.6.4 Natural factors

PM_2.5_ levels serve as an indicator of air quality, while vegetation coverage rate indicates natural environmental quality; annual average precipitation reflects the climatic conditions of the region.

The indicators are first subjected to normalization processing, followed by the selection of non-compliant influencing factors. Subsequently, the natural breakpoint method is employed to categorize the normalized data of influencing factors into integers ranging from 1 to 5. The influence value (q value) of each influencing factor on the coupling coordination degree between the aging population and older adult care facilities in Gansu Province is then calculated, where a larger q value indicates a greater impact of that factor on the coupling coordination degree. Additionally, an interactive exploration among these factors is conducted.

## 3 Results

### 3.1 Spatial and temporal evolution characteristics of the aging population

To more clearly reveal the temporal characteristics of supply–demand mismatch, this study calculated the annual growth rates of aging population and older adult care facilities for each city during 2000–2010 and 2010–2020 periods. During 2000–2010, the annual growth rate of population aged 65 and above in Gansu Province was 4.8%, while the annual growth rate of older adult care facilities was only 2.1%, with a growth speed gap of 2.7 percentage points. The regions with the most severe growth mismatch included: Jiuquan City with aging growth rate 5.9% versus facility growth rate 1.3%, creating a gap of 4.6 percentage points; Jiayuguan City at 6.2% versus 1.8%, gap of 4.4 percentage points; and Zhangye City at 5.1% versus 2.0%, gap of 3.1 percentage points. During 2010–2020, this mismatch phenomenon was somewhat alleviated but remained significant, with the provincial annual aging population growth rate at 3.9% and older adult care facility annual growth rate increased to 3.2%, narrowing the gap to 0.7 percentage points. However, mismatch problems remained prominent in some regions: Longnan City at 4.5% versus 2.8%, gap of 1.7 percentage points; and Gannan Prefecture at 4.1% versus 2.3%, gap of 1.8 percentage points. Overall, although older adult care facility construction in Gansu Province has improved, the growth speed consistently lags behind the aging process, particularly in economically underdeveloped northern and western regions where this temporal mismatch problem is more severe.

Based on the degree of population aging and the growth rate of the older adult population, the types of population aging evolution in Gansu Province are categorized into eight types (Table 2). Furthermore, ArcGIS is employed to visualize the spatial and temporal evolution of population aging.

**Table 2:** Types of population aging evolution.

Type	≥Proportion of population aged 65 and above/%	≥Average annual growth rate at age 65/%	Characteristics
Rapid aging society type	PA > 14	4 < V_PA_	Aging population is extremely high and rapidly entering the next stage of demographic aging
Rapid deep aging type	10 < PA ≤ 14	4 < V_PA_	Aging population is significant and quickly entering advanced demographic aging stage
Rapid shallow aging type	7 < PA ≤ 10	4 < V_PA_	Aging level is moderate but rapidly increasing toward deeper aging stages
Rapid non-aging type	PA ≤ 7	4 < V_PA_	Aging level is low but growth rate is rapid, indicating imminent demographic transition
Slow aging society type	PA > 14	V_PA_ ≤ 4	Aging population is extremely high but growth rate has stabilized at current level
Slow deep aging type	10 < PA ≤ 14	V_PA_ ≤ 4	Aging population is at high level but has remained relatively stable in this phase
Slow shallow aging type	7 < PA ≤ 10	V_PA_ ≤ 4	Aging level is moderate and growth rate is slow, maintaining current aging stage
Slow non-aging type	PA ≤ 7	V_PA_ ≤ 4	Aging level is relatively low and has remained stagnant with minimal growth

PA = Population Aging (proportion of population aged 65 and above, %); V_PA_ = Velocity of Population Aging (average annual growth rate of population aged 65 and above, %).

**Figure 3:**
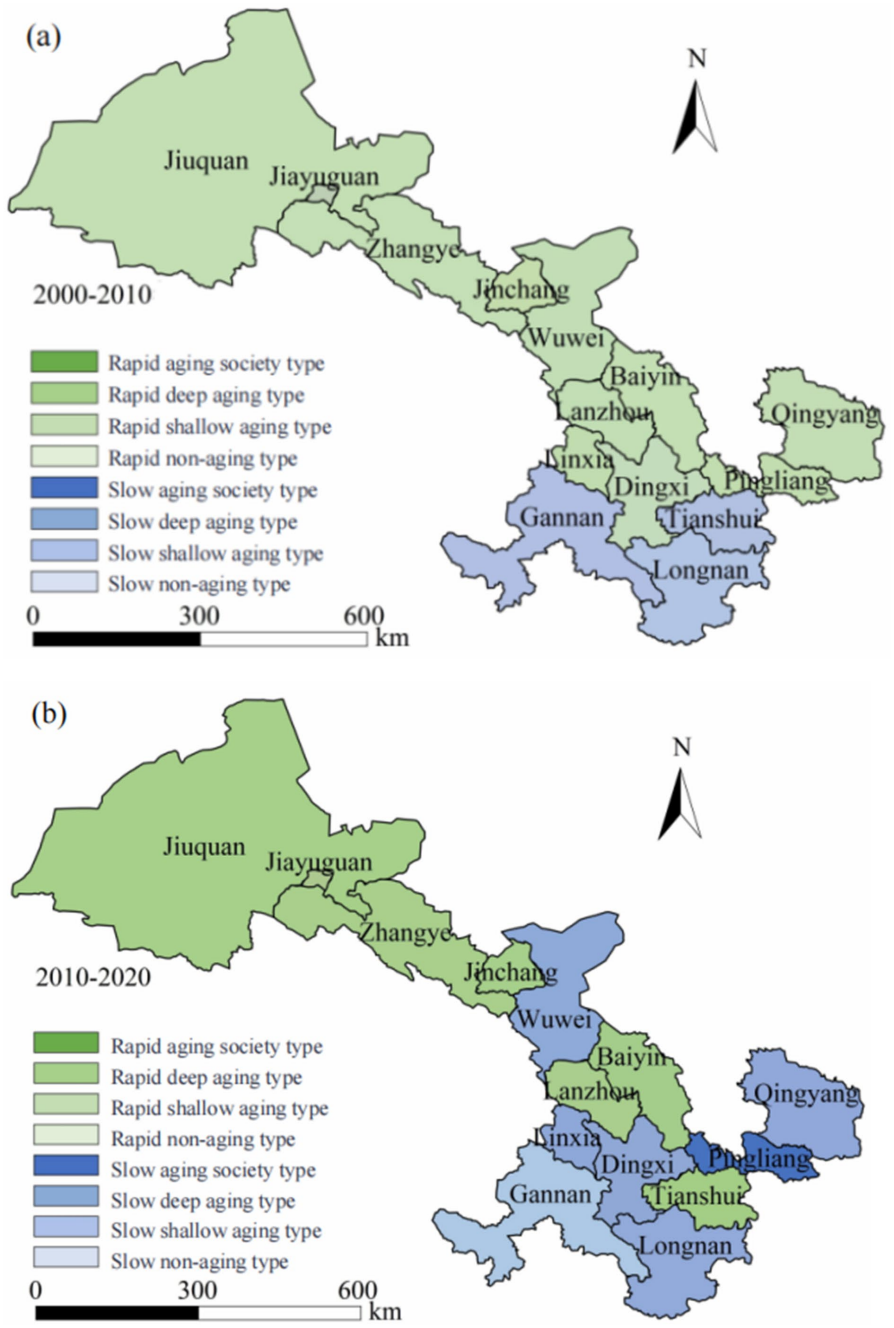
Temporal and spatial evolution of the older adult population in Gansu Province from 2000 to 2020. **(a)** Aging type distribution during 2000-2010 period; **(b)** Aging type distribution during 2010-2020 period.

The natural breakpoint method was employed to categorize the evolution of population aging in Gansu Province into eight distinct types. As illustrated in Figure 3a, during the period from 2000 to 2010, significant regional disparities in aging processes were observed across the 14 prefecture-level cities of Gansu Province. The color-coded maps clearly illustrate the varying rates and depths of aging in different areas within Gansu. Notably, the northern and central regions of Gansu are experiencing accelerated aging trends, with Cities like Jiuquan, Jiayuguan, and Zhangye belonging to the “rapid shallow aging” category, indicating that while these regions have relatively moderate aging levels (7% < PA ≤ 10%), they are experiencing rapid growth rates in their older adult populations (4% < VPA) and will soon transition to more advanced stages of population aging.

In response to the aging population in Gansu Province, it is crucial to enhance older adult care and medical services while adjusting social services and public resource allocation to address the multifaceted challenges posed by demographic shifts. In contrast, southern regions of Gansu Province, such as Tianshui City and Longnan City, exhibit a relatively slower trend toward aging. These areas, with their relatively lower proportions of older adult residents, can be categorized as regions experiencing a “slow aging process.” The blue markings on maps signify their more moderate rate of aging; although some initial signs have emerged over this decade-long span, overall changes in population structure remain mild.

The disparities in aging rates across Gansu Province are intimately tied to the economic development levels and the allocation of medical resources among different regions. In general, economically developed areas are inclined to provide more comprehensive policies to tackle challenges associated with an aging population along with better social service facilities. For instance, cities like Jiuquan and Jiayuguan, recognized for their economic advancement, have experienced accelerated aging rates, posing challenges while simultaneously prompting proactive planning in healthcare systems for elder care and in social security frameworks specifically designed for an aging society. Conversely, less economically developed regions face intensified pressures from accelerating demographic shifts toward older populations but often lack the necessary capacity and conditions to effectively address such challenges due to limited resources. This issue is particularly evident in areas with frequent migration, where the loss of young labor forces exacerbates problems related to aging demographics and further intensifies local demands for social services.

As illustrated in Figure 3b, during the period from 2010 to 2020, the aging situation in the 14 prefecture-level cities of Gansu Province underwent significant changes. The accompanying image, through its colors and markings, clearly shows that cities like Jiuquan, Jiayuguan, Zhangye, and Baiyin in northern and central Gansu are experiencing a pronounced acceleration in the aging trend. Notably, Jiuquan and Jiayuguan are marked as regions undergoing a swift transition into an aged society, indicated by green shading. The proportion of the older adult population continues to rise, posing significant challenges to social service systems. In particular, Jiuquan is identified with deep green shading, signifying its status as a “rapidly deepening aging type.” This highlights an exceptionally high level of aging in the region, consequently indicating a growing need for social resources to cater to the expanding older adult population in the coming years. Cities including Tianshui, and Dingxi are identified with green shading, all classified under the rapid aging category. The accelerated aging progression in these cities necessitates improved social resource supply to satisfy the increasing demands of the growing older adult population.

The aging trend in the southern and western regions, particularly in Longnan City, Gannan Tibetan Autonomous Prefecture, and Linxia Hui Autonomous Prefecture, has shown a notable slowdown. Notably, Longnan City and Linxia Hui Autonomous Prefecture exhibit characteristics of slow aging. Although the aging rate in these areas lags behind that of the northern and central regions, they are still experiencing a steady increase in aging trends. This necessitates proactive preparations for social older adult care and health security infrastructure.

After 2020, in specific regions such as Gannan Tibetan Autonomous Prefecture and Linxia Hui Autonomous Prefecture, a gradual yet noticeable aging trend has emerged. Despite a relatively slow growth rate of aging, the proportion of older adult people has already reached a significant level. As these areas continue to experience rising aging levels, they are likely to face increasing challenges from this demographic transition, particularly in older adult care services and healthcare provision. Slow-aging regions in southern Gansu Province, such as Longnan City, show a slower aging trend compared to other areas. This phenomenon is closely linked to lower economic development levels and specific population mobility patterns in these urban areas, marked by a substantial outflow of young individuals. As a result, the pace of aging in these regions has decelerated. However, over time, it remains essential for these areas to prepare for potential acceleration in aging within the coming years, particularly in the development of education, social security, and healthcare facilities.

### 3.2 Spatiotemporal evolution of older adult care facilities

**Figure 4:**
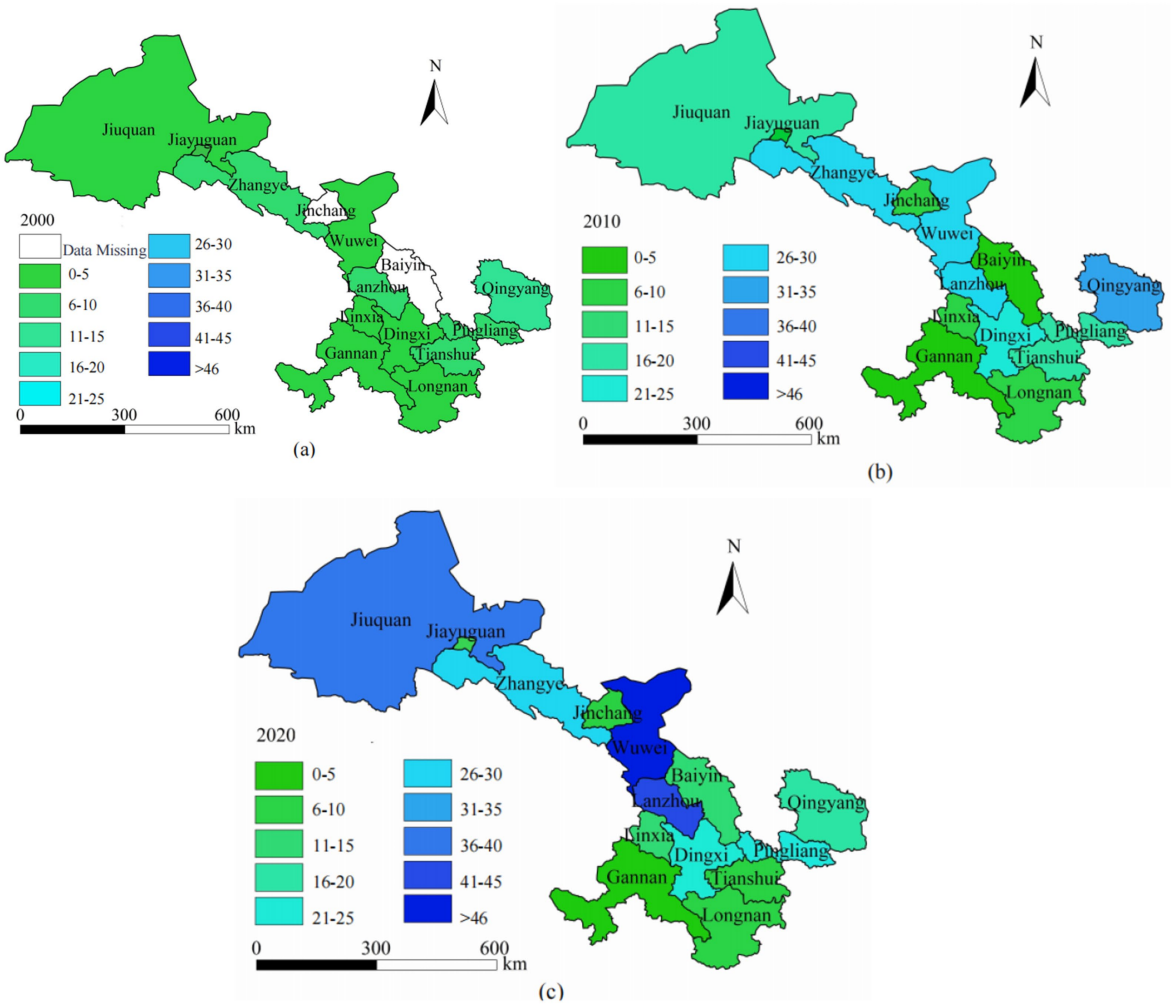
Spatial distribution map of older adult care institutions in Gansu Province from 2000 to 2020. **(a)** Distribution in 2000; **(b)** Distribution in 2010; **(c)** Distribution in 2020.

By employing the natural breakpoint method, older adult care facilities in Gansu Province have been classified into ten levels. An analysis of Figure 4a reveals notable variations in the quantity of older adult care institutions across different regions of Gansu Province in 2000. The northern and western regions, including Jiuquan City, Jiayuguan City, and Zhangye City, exhibit extremely limited availability of older adult care facilities, with most areas reporting only 0 to 5 institutions. This underscores the severe shortfall in eldercare resources due to economic constraints and lower population density, posing significant challenges in addressing the needs of an increasingly aging population. In contrast, central regions such as Lanzhou City and Pingliang City have a relatively higher number of older adult care institutions, with Lanzhou City having 18 and Pingliang City 44, according to recent data. Notably, Lanzhou, as the provincial capital, exhibits the highest concentration of these facilities, reflecting a more developed social welfare system better equipped to accommodate its aging demographic. However, even in relatively developed regions, the expanding older adult population continues to strain existing eldercare services. Furthermore, regions such as Gannan Prefecture and Qingyang City lack relevant data, suggesting incomplete records or deficiencies in their eldercare systems. Overall, this map highlights regional imbalances in older adult service provision throughout Gansu Province, particularly in economically underdeveloped areas, necessitating urgent policy intervention and increased infrastructure investment to meet the rising demands of an aging population.

The distribution of older adult care institutions across 14 cities and prefectures in Gansu Province in 2010 is illustrated in Figure 4b, revealing significant disparities in the availability of eldercare services across different regions. For instance, a report on the current status and future development trends of older adult care in Gansu states that the province had only 600 + older adult care institutions and 45,000 beds, which is significantly lower than the demand. Additionally, a registry of older adult care centers in Gansu, as outlined in the province’s older adult care service center list, highlights the uneven distribution of these facilities, with some areas having considerably more institutions than others. Compared to the year 2000, there has been a notable increase in the number of older adult care facilities. Cities like Lanzhou, Pingliang, Tianshui, and Dingxi have experienced moderate growth, with the number of institutions totaling between 26 and 30. This underscores an improvement in eldercare infrastructure within these areas. Nevertheless, regions in the north and west, including Jiuquan, Jiayuguan, and Zhangye, are still grappling with challenges due to the lack of adequate eldercare facilities; some cities report having as few as 0 to 5 institutions. This underscores the ongoing struggle of these locations to provide sufficient eldercare services. Qingyang City stands out with the highest number of older adult care institutions, totaling between 31 and 35 facilities. The overall trend suggests that Gansu Province has made significant progress in expanding its eldercare infrastructure, with a substantial increase in the number of facilities and beds, now covering 95% of urban communities and 55% of administrative villages. Nevertheless, further expansion of eldercare service facilities in underdeveloped northern and western regions remains crucial to meeting the escalating demands of an aging population in Gansu Province.

Figure 4c illustrates the distribution of older adult care institutions across Gansu Province’s 14 prefectures in 2020. For instance, in Lanzhou City, the number of older adult care facilities increased to 43, with a total of 14,290 beds, and community older adult care facilities reached 857, offering 25,710 beds by 2020. These figures demonstrate significant advancements in older adult care infrastructure across the province over the past decade. Lanzhou, the provincial capital, has a high concentration of facilities, with over 41 institutions, reflecting the well-established social care system in urbanized and economically developed areas. Central and eastern regions, such as Pingliang, Tianshui, and Dingxi, exhibit moderate growth, with institutions ranging from 26 to 40, indicating substantial progress in these areas. Nevertheless, several northern and western regions, notably Jiuquan, Jiayuguan, and Zhangye, continue to have low institution counts, ranging from 0 to 5 facilities, underscoring ongoing challenges in meeting the growing demands of the older adult population in these areas. These areas continue to struggle with providing adequate care due to economic constraints and low population density. The overall trend indicates significant advancements in older adult care facilities in more developed areas, yet underscores the necessity of sustained investment and targeted policy interventions in underdeveloped and rural areas to ensure more equitable access to older adult care services across the province.

### 3.3 Spatial and temporal evolution of the match between aging population and older adult care facilities

#### 3.3.1 Overall evaluation of the spatial–temporal evolution of the match between aging population and older adult care facilities

**Figure 5:**
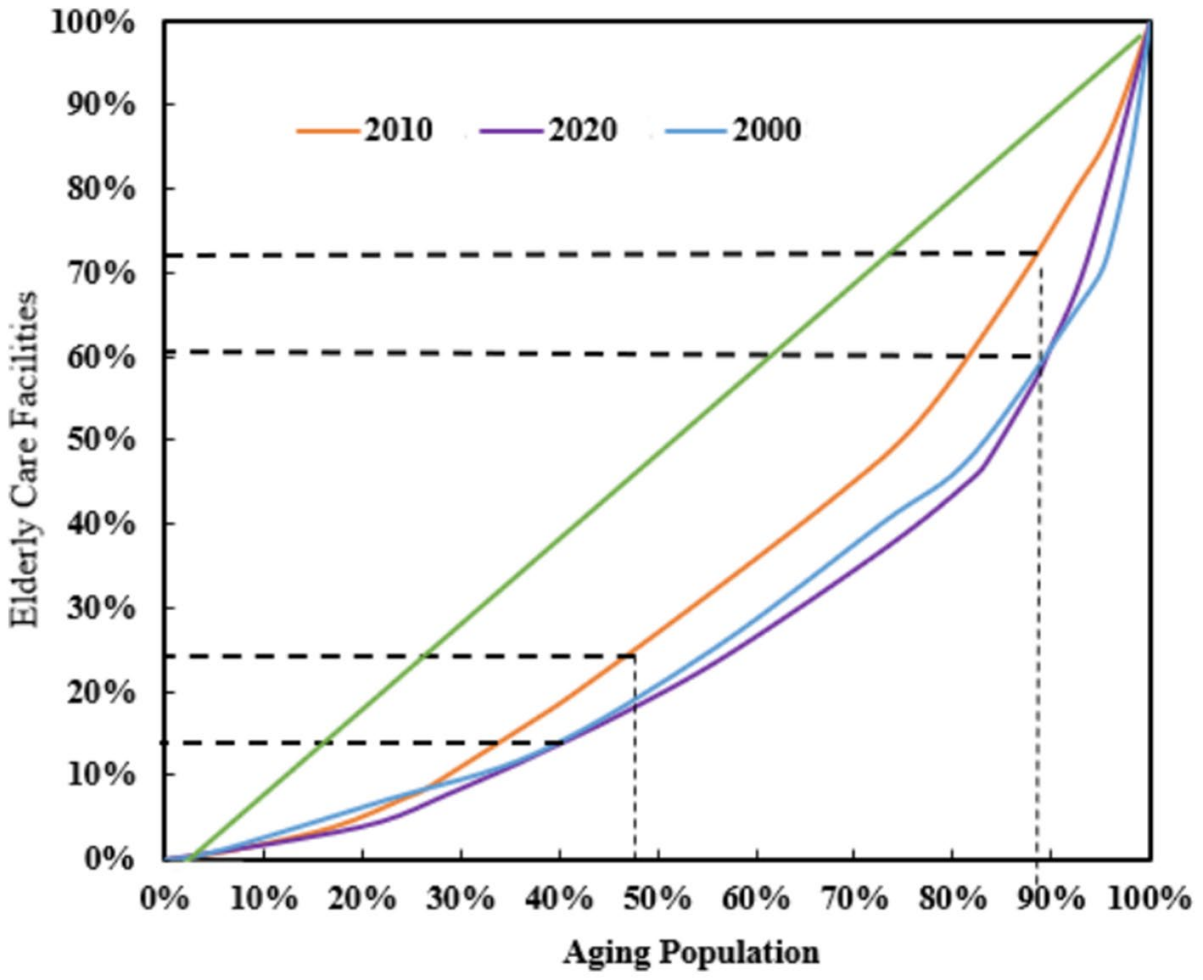
Lorenz curve of the spatial–temporal distribution of older adult care facilities in Gansu Province from 2000 to 2020.

The United Nations Development Programme classifies the Gini coefficient into five levels: a Gini coefficient ≤ 0.2 signifies a high level of equality; 0.2 < Gini ≤ 0.3 indicates moderate equality; 0.3 < Gini ≤ 0.4 suggests relatively reasonable inequality; 0.4 < Gini ≤ 0.6 denotes significant disparity; and a Gini coefficient ≥ 0.6 reflects extreme inequality. The subsequent analysis examines the relationship between the proportion of the older adult population and the availability of eldercare facilities in Gansu Province for the years 2000, 2010, and 2020. Recent data indicate a substantial shift in the availability of eldercare facilities in response to the growing older adult population in Gansu Province over the last twenty years. Figure 5, depicting the growth trend of older adult care services in Gansu Province over the past three years, shows that in 2000 (represented by the blue line), the growth rate was relatively moderate. This indicates that while eldercare facilities increased, the demand for older adult services was slow compared to the surge in the older adult population, leaving a significant disparity between the need for such services and the available provisions—thereby underscoring an unmet need in an aging society during this period. During this period, the Gini coefficient was remarkably high, falling within the range of approximately 0.4 to 0.5 (with a specific value of 0.457), indicating a distribution marked by “substantial inequality.” In contrast, by the year 2010 (represented by the orange line), there was a significant acceleration in growth, especially when the proportion of the older adult population reached approximately 40%. Nevertheless, despite certain advancements achieved during this period, the development of eldercare facilities still demonstrated lagging traits. The Gini coefficient, at 0.349, falls within the internationally recognized ‘relatively reasonable’ range, which is between 0.3 and 0.4, indicating persistent inequalities.

Moving forward to the year 2020, we observe an even steeper curve that represents rapid increases in the number of facilities. Nonetheless, these statistics lag considerably behind the growth in the older adult population, underscoring an inadequate response to their needs. Furthermore, the escalating demands on existing resources provide further proof of this issue. At this point in time, the calculated Gini coefficient escalated into ranges between 0.4 and 0.5, with a recorded value of 0.479, indicating a tendency towards “significant disparities” in distributions. This underscores the ever-widening gap between the availability of services and the actual needs of senior citizens, ultimately revealing a downward trend in matching patterns over the observed periods.

#### 3.3.2 Local characteristics of the spatiotemporal evolution

Statistical results reveal four important characteristics: First, obvious overall improvement trend is evident, as cities with coordination levels above 0.7 increased from 5 cities or 35.7% in 2000 to 9 cities or 64.3% in 2020, indicating that the matching relationship between older adult care facilities and aging population in Gansu Province has generally developed positively. Second, significant alleviation of imbalance conditions occurred, with the number of cities in severe and moderate imbalance decreasing from 4 in 2000 to 0 in 2020, demonstrating positive effects of policy interventions. Third, regional differences still exist, as despite overall improvement, 3 cities or 21.4% remain in barely coordinated or approaching imbalance states, mainly concentrated in economically underdeveloped western regions. Fourth, improvement in high-quality coordination levels is observable, as cities achieving good coordination or above increased from 3 in 2000 to 5 in 2020, indicating that some regions have established relatively comprehensive older adult care service systems (Table 3).

**Table 3:** Summarizes the distribution of coordination levels among 14 prefecture-level cities in Gansu Province across different periods.

Coordination level category	2000		2010		2020	
	Cities	%	Cities	%	Cities	%
High-quality coordination 0.9–1.0	1	7.1	1	7.1	2	14.3
Good coordination 0.8–0.9	2	14.3	3	21.4	3	21.4
Intermediate coordination 0.7–0.8	2	14.3	2	14.3	4	28.6
Primary coordination 0.6–0.7	2	14.3	3	21.4	2	14.3
Barely coordinated 0.5–0.6	1	7.1	2	14.3	2	14.3
Approaching imbalance 0.4–0.5	2	14.3	1	7.1	1	7.1
Mild imbalance 0.3–0.4	2	14.3	1	7.1	0	0.0
Moderate imbalance 0.2–0.3	1	7.1	1	7.1	0	0.0
Severe imbalance 0.1–0.2	1	7.1	0	0.0	0	0.0
Extreme imbalance 0–0.1	0	0.0	0	0.0	0	0.0

##### 3.3.2.1 Local characteristics of the spatiotemporal evolution of aging population and older adult care facilities matching relationship

According to the values of the coupling coordination degree D, the matching relationships between the older adult population and older adult care facilities can be categorized into ten distinct types: a value of D in [0, 0.1] indicates extreme imbalance; a value in [0.1, 0.2] signifies severe imbalance; a range of [0.2, 0.3] represents moderate imbalance; a value in [0.3, 0.4] denotes mild imbalance; a range of [0.4, 0.5] is classified as approaching imbalance; values from [0.5, 0.6] indicate barely coordinated conditions; the interval [0.6, 0.7] reflects primary coordination; a range of [0.7, 0.8] suggests intermediate coordination; a value within [0.8, 0.9] demonstrates good coordination; finally, [0.9, 1.0] represents high-quality coordination.

**Figure 6:**
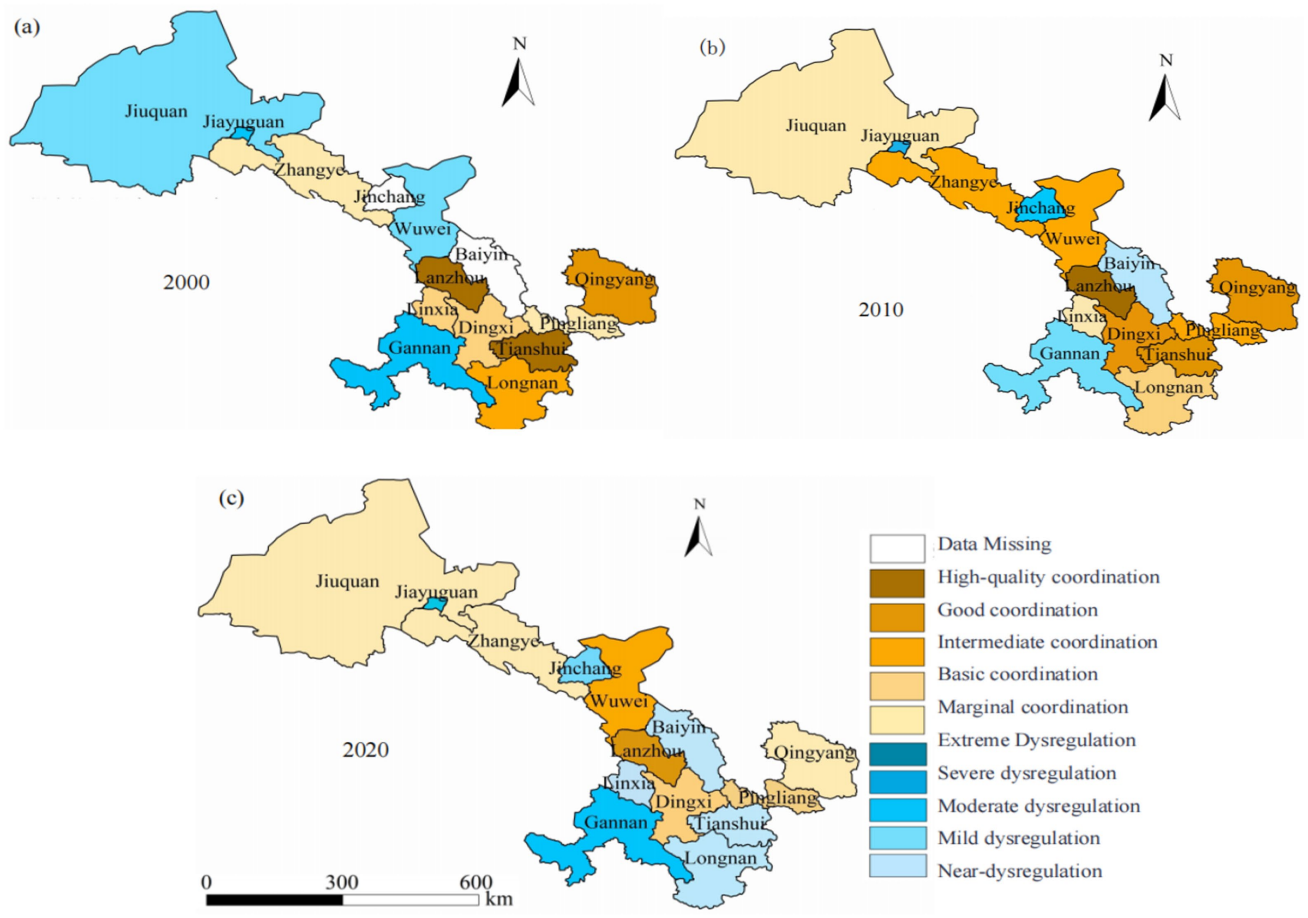
Temporal and spatial evolution of the adaptability between the older adult population and older adult care facilities in Gansu Province from 2000 to 2020. **(a)** Coupling coordination degree in 2000; **(b)** Coupling coordination degree in 2010; **(c)** Coupling coordination degree in 2020.

Figure 6a illustrates the coupling coordination degree (D) between the aging population and older adult care facilities across various cities in Gansu Province in the year 2000. Overall, there are significant regional disparities in the alignment between older adult care facilities and the demands of an aging population within Gansu Province. Some areas exhibit good coordination, while others face considerable shortages in eldercare services. Specifically, Lanzhou City demonstrates the highest level of coordination between its older adult care facilities and aging population needs, characterized by excellent coordination (D > 0.9), indicating a well-developed eldercare service system. Cities such as Pingliang and Dingxi show good coordination (0.8 ≤ D < 0.9); although their older adult care facilities are relatively sufficient, there remains room for improvement. In contrast, Tianshui and Linxia display moderate coordination (0.7 ≤ D < 0.8), suggesting that further enhancements to service infrastructure are necessary. Zhangye and Jiayuguan are categorized under primary coordination (0.6 ≤ D < 0.7), indicating a delay in the development of older adult care facilities, which urgently requires additional investment. Regions such as Jiuquan exhibit a severe imbalance (D < 0.5), characterized by insufficient eldercare resources that cannot fulfill the demands of their aging populations; these areas require focused attention and support. Gansu Province must achieve balanced resource allocation among regions concerning eldercare facility construction, particularly emphasizing improvements in economically disadvantaged areas to effectively address future challenges posed by an increasing aging population.

Figure 6b illustrates the coupling coordination degree (D) between the older adult population and pension facilities across various cities in Gansu Province in 2010, indicating a gradual increase in the demand for older adult care services and the expansion of pension facilities, with notable improvements in the number of older adult care institutions and beds available per thousand older adult individuals. In Gansu Province, there has been a noted improvement in the alignment between pension facilities and the demands of an aging population, yet significant regional disparities persist, with challenges such as inadequate infrastructure, a shortage of facilities, and a lack of comprehensive services. Lanzhou City showcases a well-coordinated approach to older adult care, with a robust network of pension facilities that adeptly cater to the needs of its aging population. In contrast, economically underdeveloped cities such as Pingliang and Dingxi demonstrate effective coordination, signifying advancements in the development of their pension infrastructure. Conversely, Zhangye, Jiayuguan, and Jiuquan exhibit lower degrees of coordination, categorized as either primary coordination or moderate imbalance, implying inadequate addressing of the challenges associated with demographic aging and significant service deficiencies. Furthermore, specific regions such as Tianshui and Linxia exhibit a low degree of matching between pension facilities and their aging populations, indicating mild imbalances in these regions. In summary, while there has been some improvement in the coverage of pension facilities within Gansu Province by 2010, substantial regional differences remain evident. Especially in less economically developed areas, there is an urgent need for investment and policy support in pension facility construction to align with demographic changes.

From Figure 6c, it can be observed that the matching degree between older adult care facilities and the aging population in Gansu Province improved in most regions by 2020; however, significant regional disparities still exist. Lanzhou City continues to demonstrate a high level of coordination, indicating that its older adult care facilities have adequately met the needs of the aging population and that its older adult service system is relatively well-developed. Regions such as Pingliang City and Dingxi City exhibit good coordination; despite some growth in older adult care facilities, further development is still required to address the demands arising from demographic aging. Conversely, regions such as Linxia Prefecture and Tianshui City demonstrate moderate levels of coordination, implying that although their older adult service systems have seen some development, they have not adequately coped with the pressures stemming from population aging. Relatively speaking, certain western and northern regions—such as Jiuquan City and Jiayuguan City—exhibit lower levels of coordination. Notably, Jiuquan City displays severe imbalances; the construction of older adult care facilities in these areas has failed to keep up with the aging population trends, leading to significant service deficiencies. Overall, despite improvements in the coherence of older adult care facilities in Gansu Province from 2000 to 2010 through to 2020, notable inter-regional differences remain evident. Particularly in economically disadvantaged areas, there is a pressing need for increased investment in eldercare facility construction and policy support.

### 3.4 Analysis of factors influencing coupling coordination degree

#### 3.4.1 Single factor detection result

The analysis in Table 4 reveals that all influencing factors passed the 5% significance level test, indicating their significant impact on the coupling coordination degree between the aging population and older adult care facilities in Gansu Province. From the perspective of determining power (*q* values): (1) The base of population aging is the primary determinant with a q value of 0.357. (2) Population density serves as a secondary factor with a q value of 0.351, while the older adult dependency ratio ranks sixth at 0.155. This demonstrates that population density and the aging base of each city are pivotal factors influencing the degree of coupling coordination, which is further affected by regional economic, social, and natural conditions. (3) The total index of digital inclusive finance has a relatively high impact on coupling coordination with a q value of 0.326, ranking third. Recently, rapid urbanization and economic growth in China have resulted in a heightened demand for older adult care facilities among the aging population.

**Table 4:** Analysis of influencing factors on coupling coordination in older adult care services degree.

Classification	Indicator	Decision-making ability (*q* value)	Significance level (*p*-value)	*q* sorting
Population factors	Population density	0.351	0.035	2
	Population aging base	0.357	0.027	1
	Old-age dependency ratio	0.155	0.034	6
Economic factors	General public expenditure	0.154	0.021	7
	Digital inclusive finance index	0.326	0.017	3
Social factors	Number of older adult care facilities per thousand seniors	0.221	0.032	5
	Number of healthcare beds per thousand older adult individuals	0.309	0.025	4
Natural factors	Vegetation coverage rate	0.121	0.033	10
	Average annual precipitation	0.125	0.011	9
	PM_2.5_	0.146	0.015	8

From the perspective of four influencing factors, population and economic factors are the dominant influences on the coupling coordination degree between the older adult population and pension service facilities in Gansu Province. Among them: (1) Population factors exert the strongest influence on the coupling coordination degree, serving as a fundamental determinant. To combat aging trends, attracting migrant labor is crucial for boosting birth rates. (2) Economic factors rank second in their decision-making power; notably, the total index of digital inclusive finance has a more significant imp act than general public financial expenditure. This indicates that economic development has improved digital inclusive finance services in rural or poorer areas, positively affecting coupling coordination. Furthermore, imbalances in regional economic development lead to higher coupling coordination degrees in provincial capitals, in contrast to prefecture-level cities. (3) Social factors exert minimal influence on coupling coordination; Specifically, the ranking of healthcare beds per thousand older adult individuals stands at fourth, while available eldercare institutions occupy fifth place, with decision-making powers of 0.246 and 0.218, respectively. With the improvement of living standards and healthcare technology, social factors will play an increasingly significant role in affecting coupling coordination. (4) Natural factors have the least impact on coupling coordination; however, from a macro perspective, a favorable natural environment can enhance life expectancy and subsequently influence the relationship between older adult populations and pension service facilities.

#### 3.4.2 Analysis of factor interaction effects

The spatial distribution of the older adult population and the provision of older adult care facilities are crucial for understanding the interaction effects between factors. For instance, the study on Yangzhou’s older adult population and care facilities highlights the mismatch between the distribution of older adult population types and the supply of care facilities, indicating a need for more adaptable and comprehensive care services.

**Figure 7:**
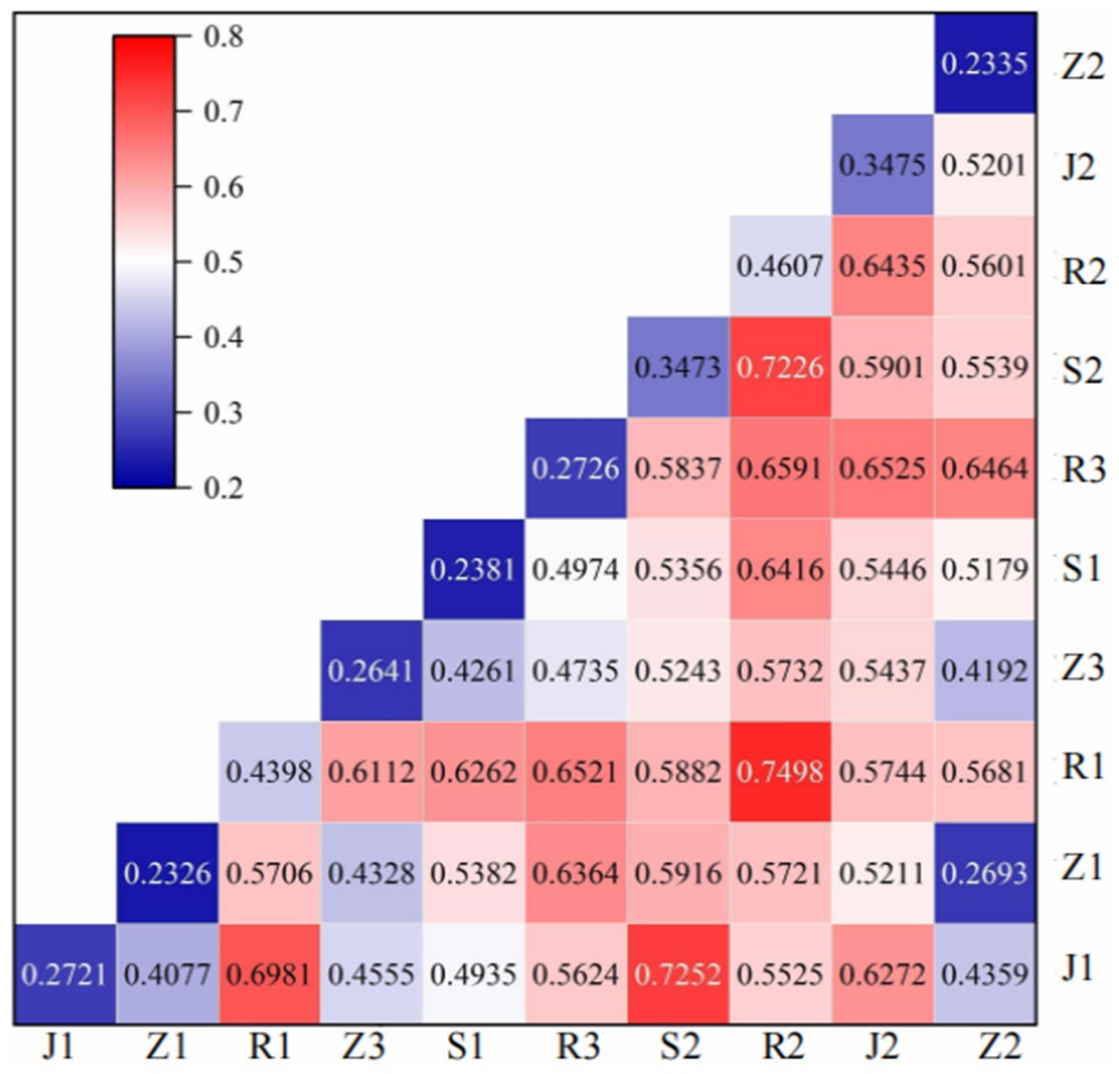
Analysis of interaction detection results for influencing factors on coupling coordination degree. Population density (R1); population aging base (R2); old-age dependency ratio (R3); general public expenditure (J1); digital inclusive finance index (J2); number of older adult care facilities per thousand seniors (S1); number of healthcare beds per thousand older adult individuals (S2); vegetation coverage rate (Z1); average annual precipitation (Z2); PM_2.5_ (Z3).

The analysis in Figure 7 indicates significant variations in the impact of individual factors on the degree of coupling coordination, as evidenced by similar studies on urbanization and eco-environment, resource–population–industry systems, digitalization and higher education, and agricultural carbon emission efficiency and green finance. By employing geographic detectors to investigate the interactions among various influencing factors within the study area, we identify that the top five pairs exhibiting high q values for coupling coordination are as follows: population density ∩ aging base (0.7498) > general public fiscal expenditure ∩ number of healthcare beds per thousand older adult individuals (0.7252) > aging base ∩ number of healthcare beds per thousand older adult individuals (0.7226) > population density ∩ general public fiscal expenditure (0.6981) > aging base ∩ older adult dependency ratio (0.6591); these results indicate that 76.4% of indicator interaction combinations demonstrate a nonlinear enhancement relationship, while 23.6% exhibit a dual-factor enhancement relationship, with no independent factors identified as having an isolated effect. Notably, the interaction between population density and aging base exerts the most substantial influence on regional coupling coordination levels, indirectly highlighting that demographic factors are essential for understanding aging dynamics. Next, we consider the dual-factor interaction between public fiscal expenditure and the availability of healthcare beds per thousand older adult individuals. From a holistic perspective, there is a notable correlation between the number of older adult care facilities and various socio-economic and environmental variables. Recent studies have observed significant positive correlations with indicators such as the aging population ratio, the old-age dependency ratio, and the number of older adult care institutions per thousand older adult individuals, with correlation coefficients reaching 0.6981 and 0.7252. This suggests that the distribution of older adult care facilities is closely related to the degree of population aging.

Furthermore, a negative correlation (−0.5744) between PM_2.5_ levels and population density indicates that areas with higher population density may experience more severe air pollution, thereby potentially impacting residents’ health negatively and increasing their demand for older adult care facilities. The positive correlation (0.6272) between fiscal expenditure and the number of older adult care institutions suggests that higher public financial investment could lead to the development of more eldercare services, thereby enhancing service capacity for an aging population. Furthermore, a robust correlation coefficient of 0.6435 between the Aging Foundation Index and the Digital Financial Inclusion Index indicates that regions experiencing a higher degree of aging are inclined to depend on digital technologies to deliver essential financial services to the older adult. The data indicate that the relationship between aging populations and associated socio-economic factors is complex yet intimate; thus, policymakers should consider incorporating multidimensional factors.

Interaction significance test results show that among 45 variable interaction combinations, 42 combinations passed the 5% significance level test, with 3 combinations having significance levels between 0.05–0.1. Among these, the interaction between population density and population aging base has the highest significance with *p*-value less than 0.001, indicating extremely high statistical reliability of this interaction relationship. The interactions between fiscal expenditure and healthcare beds, and between population aging base and healthcare beds also reached significance levels of *p* < 0.001. These highly significant interactions provide reliable statistical basis for policy formulation.

The coupling coordination degree between the aging population and older adult care service facilities is influenced by various interacting factors, exhibiting significant dual-factor enhancement and nonlinear enhancement effects. Among these factors, demographic and economic variables stand out as the primary drivers influencing the degree of coupling coordination. Among demographic factors, the increasing base of population aging exerts the most significant influence, serving as a fundamental determinant. Furthermore, economic considerations also play a significant role. Rural and relatively impoverished areas have seen benefits from enhanced digital inclusive financial services, positively affecting the coupling coordination degree. However, there exists an imbalance in regional economic development disparities. Social factors exert minimal influence on the coupling coordination degree, while natural factors have the weakest effect. Furthermore, it is crucial to recognize the interdependence among these influencing factors; employing geographic detectors can enhance the extent of each factor’s impact on the coupling coordination degree.

## 4 Discussion

The theoretical contributions of this study are primarily reflected in the practical application and extension of spatial justice theory in underdeveloped regions. First, we validated the applicability of spatial justice theory under resource-constrained environments, finding that traditional spatial justice theory needs localized adjustments combined with local economic development levels and geographical conditions. The empirical results from Gansu Province indicate that in underdeveloped regions, absolute spatial equality is not always the optimal choice, requiring a balance between spatial equity and economic efficiency. Second, we enriched the empirical content of health equity theory by revealing the compound influence mechanism of geographical, economic, and demographic factors on health resource accessibility through quantitative analysis. Finally, we extended the application of social resource allocation theory under multi-constraint conditions, proposing resource allocation optimization strategies applicable to topographically complex and economically underdeveloped regions, providing new empirical evidence for further development of related theories.

This study conducts an in-depth analysis of the spatiotemporal evolution of the aging population and older adult care facilities in Gansu Province, with a focus on the period from 2000 to 2020, updating the findings with the latest data available as of 2023. It reveals significant disparities in the aging process and the construction of older adult care facilities across different regions within the province. The findings indicate that the northern and central regions of Gansu (such as Jiuquan, Jiayuguan, and Zhangye) are experiencing a rapid aging process, with a notable increase in the proportion of older adult individuals over the past two decades, thereby entering a phase characterized as a “rapidly aging society.” In contrast, southern and western areas, including Longnan, Linxia, and Gannan, exhibit a slower pace of aging, reflecting characteristics typical of a “slowly aging society”. Through analyzing these regional differences, it becomes evident that the speed of demographic aging is closely related to multiple factors such as economic development levels, allocation of social resources, and population mobility.

From the perspective of the distribution of older adult care facilities, regions with relatively developed economies are leading in the construction of such facilities, thereby partially meeting the growing demand from the aging population. However, in the northern and western parts of Gansu Province, particularly in economically lagging areas like Jiuquan, Jiayuguan, and Zhangye, there is a noticeable delay in the development of older adult care facilities. This shows that the rapid increase in the older adult population in China is outpacing the development of eldercare services, exacerbating aging-related issues as the supply significantly falls short of the growing demand. Similar trends are evident across the country. Economically underdeveloped areas face a particularly acute shortage of eldercare service facilities, posing a major challenge for an aging society. The findings of this study have important implications for other developing countries. According to the WHO Global Report on Aging and Health (2023), regions with high topographical complexity, low economic development, and accelerating aging commonly face spatial allocation challenges for older adult care facilities. UN-Habitat research indicates that facility accessibility in mountainous areas of developing countries is generally 40–50% lower than in plains. Therefore, the hierarchical service system and terrain-corrected accessibility model proposed in this study have cross-national promotion potential, particularly suitable for topographically complex developing regions such as South Asian mountainous areas and Latin American plateau regions.

This study quantitatively examines the relationship between the aging population and older adult care facilities across various regions of Gansu Province, employing coupling coordination degree (*D* value) and Gini coefficient analyses. The research is underpinned by data indicating that as of last year, Gansu had 2.1956 million individuals aged 65 and above, representing 8.50% of the total population. The province is home to 510 older adult care institutions with a combined total of 27,260 beds, yielding an occupancy rate of 55.83%. However, infrastructure to address population aging in certain areas remains inadequate; specifically, medical facilities and staffing levels within older adult care institutions are insufficient. While cities such as Lanzhou have made significant progress in developing older adult care service facilities, largely due to advancements in digital finance and social security systems—a substantial gap persists between the aging population and the availability of these resources throughout Gansu Province. This discrepancy is particularly pronounced in economically disadvantaged northern and western regions where the pace of aging has accelerated; however, development of nursing facilities has not kept pace with this demographic shift, resulting in a persistent issue characterized by a “supply–demand imbalance.” Factors influencing the relationship between the aging population and nursing facilities in Gansu Province include population density, economic development level, age demographics, and fiscal investment. Especially in northern and western regions, insufficient financial support and inadequate social security systems have hindered the construction of older adult care facilities, adversely affecting both the quality of life and social security for the aging population. Conversely, the emergence of digital finance, particularly in rural areas, presents an opportunity to mitigate the shortage of older adult care services by improving financial service accessibility for older adults. However, despite its beneficial role in alleviating pressures associated with aging populations, significant disparities exist in digital finance penetration and coverage across different regions. Notably, some remote areas continue to experience a lack of comprehensive integration of digital finance, which still fails to adequately address issues arising from insufficient older adult care facilities.

Compared to existing research both domestically and internationally (22, 23), this study’s innovation lies in its detailed spatiotemporal analysis that quantitatively assesses the relationship between the aging population and older adult care facilities across different regions of Gansu Province. By integrating the coupling coordination degree and the Gini coefficient, this study provides more scientifically robust data support (24). Previous studies have predominantly focused on macro-level analyses, with limited systematic exploration of the relationship between population aging and the development of older adult care facilities from a regional perspective (25). This is particularly true for underdeveloped areas, where issues related to eldercare services remain inadequately addressed (26). This study fills this research gap by introducing a refined analytical framework, grounded in social resource theory, to enhance the understanding of older adult care satisfaction.

The detailed analysis of Gansu Province’s aging population, as presented in the references, offers significant practical implications for policy formulation in the region and similar areas. In response to the rapid aging process in the northern and central areas of Gansu Province, local governments have been actively enhancing the construction of older adult care facilities. As of 2019, the province had achieved coverage of 95% of urban communities and 55% of administrative villages with these services. Furthermore, by the end of 2022, additional facilities were built, achieving full coverage in urban streets. These efforts reflect a significant commitment to improving the quality of life for the older adult in Gansu. In light of the aging trends observed in regions such as Gansu Province, where the older adult population is growing and the burden of social care is increasing, it is imperative to develop social security policies that are region-specific, taking into account the unique aging patterns and economic conditions. For economic reasons, it is crucial to ensure the sustainability of these policies by considering local fiscal capacities. Economically disadvantaged southern areas, despite currently facing less pressure from aging populations, are anticipated to encounter a worsening of this issue in the future. Therefore, it is imperative that these regions proactively plan and construct older adult care facilities to prevent potential imbalances between supply and demand.

Promoting digital finance plays a positive role in alleviating economic pressures on older adults and improving eldercare services (27), particularly in rural and underdeveloped areas (10). The government should increase support for digital financial services to facilitate their adoption among senior citizens (28). This would provide older individuals with more convenient access to social welfare programs and eldercare services while reducing the burden associated with traditional care giving methods (29). Furthermore, as technology continues to advance rapidly, emerging solutions such as smart eldercare systems and telemedicine hold great promise for enhancing eldercare services, especially in resource-scarce regions. Future research should focus on how these new technologies can be leveraged to improve service efficiency.

This study has limitations regarding spatial scale. Our analysis is based on prefecture-level administrative units, and this larger spatial scale may mask significant heterogeneity within cities. Taking Lanzhou City as an example, the density of older adult care facilities in urban core areas such as Chengguan District is significantly higher than in peripheral areas such as Yuzhong County, while distribution patterns of older adult population, economic conditions, and transportation accessibility also vary considerably. Prefecture-level averaged analysis fails to capture such intra-urban spatial differentiation characteristics and may underestimate service gaps in certain areas or overestimate overall coordination levels. Future research recommendations include adopting more refined spatial scales, such as county-district level or even street-community level analysis, to more accurately identify spatial hotspot areas of older adult service supply–demand mismatches. Meanwhile, it is recommended to combine urban planning and land use data to deeply analyze eldercare service allocation characteristics in different functional zones within cities, providing more targeted scientific basis for precise facility layout planning. Additionally, the dynamic impact of urbanization processes on older adult population spatial distribution should be considered, establishing multi-scale analytical frameworks that can reflect intra-urban complexity.

The research primarily focuses on macro factors such as population density and economic development that influence the construction of older adult care facilities. However, it lacks sufficient exploration of social and cultural factors, including the diverse perceptions of aging across regions, the significance of family caregiving, and the impact of these cultural differences on older adult care needs. Significant disparities in cultural backgrounds and elder care demands across different regions may influence both the acceptance and effectiveness of older adult care facilities. Future research should incorporate diverse cultural contexts and social structures to explore the multifaceted role of social and cultural factors in aging-related issues. This approach will not only enhance our understanding of these factors but also contribute to the development of targeted policy recommendations that address the diverse needs of aging populations. In the policy formulation process, it is necessary to fully consider the diverse eldercare preferences and behavioral characteristics of older adult individuals. Existing research shows that Chinese older adult generally prefer aging in place, with institutional care often being the last choice. In Gansu Province, where traditional culture is well-preserved, this preference is even more pronounced. Therefore, simply increasing the number of institutional care facilities may not effectively meet actual demands and may even cause resource waste. Policy recommendations should start from the demand side, constructing a multi-level service system combining home care, community care, and institutional care. Specifically: First, strengthen home care support systems through door-to-door services, remote monitoring, family care training, etc., to meet most older adult people’s preferences for aging in place; Second, improve community eldercare service functions by building day care centers, community kitchens, health management stations, etc., to provide convenient nearby services for older adult people living at home; Third, optimize institutional eldercare service positioning to focus on providing professional services for disabled, cognitively impaired older adult and those without family care conditions, avoiding blind scale expansion. Additionally, it is necessary to deeply understand actual needs of older adult people in different regions and groups through demand surveys and preference analysis, achieving precise matching between supply and demand sides to avoid planning mismatches and resource misallocation caused by ignoring user preferences.

## 5 Conclusion

The quantitative analysis results of this study reveal key characteristics of spatiotemporal evolution of population aging and older adult care facilities in Gansu Province. Regarding aging processes, the proportion of population aged 65 and above in Gansu Province increased from 8.9% in 2000 to 17.03% in 2020, with an annual growth rate of 4.2%, entering the stage of deep aging. Regarding facility construction, the number of older adult care institutions increased from 156 in 2000 to 510 in 2020, with a growth rate of 227%, but still lower than the 341% growth rate of aging population. Regarding supply–demand matching, the Gini coefficient showed a U-shaped trajectory: declining from 0.457 in 2000 to 0.349 in 2010, then rising to 0.479 in 2020, indicating that spatial matching equity first improved then deteriorated. Regarding coordination levels, the proportion of cities with coordination levels above 0.7 increased from 35.7% in 2000 to 64.3% in 2020, an increase of 28.6 percentage points; meanwhile, cities in imbalanced states decreased from 28.6% in 2000 to 7.1% in 2020, a reduction of 21.5 percentage points. Regarding growth rate comparisons, the growth rate gap between aging population and older adult care facilities reached 2.7 percentage points during 2000–2010, narrowing to 0.7 percentage points during 2010–2020, but supply lag problems persist. Regarding spatial differentiation, aging growth rates in northern and central regions generally exceed 5%, while older adult care facility growth rates are mostly below 3%, highlighting prominent spatial mismatch problems.

The analysis of the matching relationship indicates that, despite an overall rise in the quantity of older adult care facilities, their geographical distribution does not align with the spatial distribution of the aging population. Variations in the Gini coefficient indicate that the equity of older adult care resource allocation has not been consistently improved. Moreover, the coupling coordination analysis demonstrates that Gansu Province’s overall matching degree has consistently remained at a comparatively low level, with significant regional disparities. Further investigation identifies key factors influencing the coordination between population aging and older adult care facility matching: population density, economic development, fiscal investment, and levels of digital inclusive finance. Notably, the interaction between population density and aging fundamentals is particularly pronounced (*q* = 0.7498), indicating that the degree of population concentration directly determines the supply–demand relationship for older adult care resources. Additionally, areas with advanced economic development enjoy substantial fiscal support and comprehensive older adult care facility construction. On the contrary, economically less developed regions confront issues like inadequate facilities and inefficient resource allocation, which further intensify the supply–demand imbalance in eldercare services.

Based on the research findings, Gansu Province is recommended to adopt a regionally differentiated approach in planning older adult care facilities, aiming to improve the alignment and equity of older adult care resources. In the resource-scarce northern and western regions, increased investment in older adult care services is needed, along with the optimization of infrastructure development and the establishment of a more flexible and efficient older adult care service system tailored to the population mobility characteristics. Concurrently, steps must be taken to foster the merging of digital inclusive finance with older adult care services, thereby enhancing accessibility for the older adult population residing in remote regions. Moreover, the collaboration between government entities and diverse societal sectors should be fortified by enhancing policy support, optimizing financial allocations, and incorporating market mechanisms. This approach seeks to improve the coordination among older adult care facilities and demographic aging trends while ultimately achieving balanced development and sustainable optimization of older adult care services within regions.

## Data Availability

The original contributions presented in the study are included in the article/supplementary material, further inquiries can be directed to the corresponding author.
